# Sir Andrew Clark

**Published:** 1893-11-11

**Authors:** 


### The Hospital

An Institutional Journal of

Science, Medicine, Hygiene, Nursing, and News.

*For Hospitals, Asylums, Infirmaries, Dispensaries, Medical Practitioners, Students, and Nurses.*

Vol. XV.—No. 372.] [Nov. 11, 1893.

### Sir Andrew Clark.

It is difficult to realise that Sir Andrew Clark is dead. A month ago he seemed to have before him the prospect of many years of life, and even of active work. Few who knew him well will doubt that if he had worked less arduously he would have lived longer to serve and sweeten his generation. There are many physicians and surgeons still surviving, and some in the full tide of active practice, who were famous before Sir Andrew Clark began to be generally known. So quickly does a too ardent fire burn itself out.

Sir Andrew Clark, as we have said before in these pages, was something of what Carlyle would have called "a hero," and in the capacity of hero he had his worshippers. He began life modestly. His parents, we believe, belonged to the farming class, though his father is said, for a time at any rate, to have followed the profession of medicine. In youth the future physician was a country doctor's apprentice, and he had for his fellow-apprentice William Aitken, who afterwards became Sir William Aitken, the head of Netley Hospital.

It was by virtue of what was in him and nothing else that Sir Andrew Clark became what he was and achieved what he did. When he applied for the post of assistant physician to the London Hospital, as he himself told the present writer, he did it with fear and trembling. He was poor, he had no powerful friends, and he was delicate. One of the members of the committee of appointment, pleased with his testimonials and pitying his pallor, said half contemptuously, "Let us give it"—the appointment—"to this poor Scotch fellow; he wont want it long." How he kept the appointment; how he exalted it; how he made his hospital and himself famous; how he won the esteem, the confidence, the love, and the admiration of generation after generation of medical students has long been known to them, and will now be made known to all the world.

Many years ago Mr. Gladstone said to a Scotch friend of the present writer's, "When I need a doctor, I call in a fellow countryman of yours, Dr. Andrew Clark. I have the greatest possible confidence in him." It was when the public understood that Mr. Gladstone's powerful and acute intellect had selected Dr. Clark for its confidence, that the tide of professional prosperity began to flow; and it proved to be a tide which, though long in rising, never fell again. It continued to be a full tide to the last day of Sir Andrew Clark's health. Sir Andrew and Mr. Gladstone, at any rate during the last few years, had come to be regarded by the public as in some sense twins. All the world has admired and delighted in Mr. Gladstone's wonderful physique and intellectual force and youthfulness; and all the world, by the most natural transition of thought, has set its eyes upon Sir Andrew Clark as in some sort the earthly providence which has preserved to Mr. Gladstone the astonishing vigour, versatility, and freshness of his age. For although Mr. Gladstone's popularity as a statesman may have lately waned with large numbers of his fellow countrymen, there is not one of them but takes unalloyed delight in his venerable and majestic form, and in the ardour and energy of his intellectual work. Just as in the last century the literary man became most speedily famous who deserved and found a judicious and trusted patron, so in these times the fame of the great statesman raised to fame the physician who deserved and secured his confidence.

The fame of Sir Andrew Clark is the fame of the physician. A specialist he could have been, and he would have been a successful one; a man of science he could have been, and he would have achieved fame. But he chose to be a practising physician, and he gave his whole soul to therapeutics. A pathologist he was, and a physiologist; a diagnostician, too, who added to an admirable capacity the merit of infinite pains and patience. But it was as a healer, as a man who devoted his unparalleled energies to the art of cure, or of comfort and relief, that he became both famous and truly great. The end and the means he never confused, and he never saw either the one or the other out of proportion. The end was the cure, or, if that were not possible, the comfort of the patient, and to that everything else was sacrificed. This, we maintain, is the highest work to which the physician can devote himself. To this science of every kind, great as it is, must be subordinate. The end must always be greater than the means, however great the means in themselves may be. The great end of human happiness and well-being was that to which Sir Andrew Clark devoted all his powers of body, mind, and soul.

Sir Andrew Clark came to honour. He held in succession every office which the Royal College of Physicians had it in its power to bestow. As lecturer, as examiner, as censor he was equally thorough, equally competent, and equally esteemed. The highest of all medical honours came to him in 1888, when he was elected by his colleagues, in succession to Sir William Jenner, President of the Royal College of Physicians. Five years before he had received the honour of a baronetcy at the hands of the Queen.

So much for the physician and his life's work. The world at large will long miss and remember Sir Andrew Clark. For his friends, and they are many, and for those amongst them who had the high privilege of his personal affection, the loss is irreparable. Sir Andrew Clark, from the day when The Hospital first appeared, has been one of its warmest supporters and most interested readers. He was always willing to help in a critical emergency by sound advice and counsel, and on more than one occasion he contributed to these columns articles of weight and authority. As a colleague, as a dear personal friend, as a man, we feel we may not hope to look upon his like again. For a long time past, on the last Sunday of the old year, we used to meet our departed friend in the intimacy of close personal friendship, and at such times Sir Andrew Clark was seen at his best. His vitality was remarkable; his wide knowledge, extensive reading, and absorbing interest in affairs made him one of the most delightful and attractive of companions. As a friend he was full of sympathy, ever ready to help the weak and humble, and indeed all who had any right to consult him, and the better he was known the more he was beloved.

We know that Sir Andrew Clark looked forward as we looked forward for him to many years of useful life, wherein he would be able by the light of his experience to help movements of the greatest importance to the well-being of the people and especially of the poor. But, alas! he has gone to his rest, struck down in the midst of his busy life, taken from us with a suddenness which makes his loss all the more real and hard to bear. Still, we cannot wish for him a different fate from this, because we know that it was his desire to die at his post. Only a few weeks ago, when speaking of the broken holiday which resulted from the great pressure upon him during the last few months of his life, he remarked that, after all, there was nothing to regret, for his work and life in London were the very breath of his nostrils and the happiest and brightest days in all the year. Knowing and loving him as we did, we are confident that the sense of loss created by his death will deepen and widen and extend with time. Yet we cannot but rejoice that to the last moment, when health was his, he was permitted to devote his whole strength to the task of alleviating the sufferings of others, ever regardless of his own health and of the increasing demands which those claims made upon his strength, until he, who had built up and conserved the strength of a multitude, fell a victim to the overstrain which a high sense of duty caused him to disregard in his own person. He is gone to his rest, a great physician and a good man. Surely no better epitaph could be won or desired by the very best of the race.

As a man of science Sir Andrew Clark was eminent, as a physician he was unique; and it was beginning to be seen by many that he aspired to give a tone to the youth and manhood of the period, a tone of strenuousness, of work idealised, of hope in a long immortality, when man shall be separated from his mortal accidents, and shall live as a pure and high intelligence expanding through the aeons of the unending future. His fullest energies he gave to his medical study and work; but at least his leisure he devoted to those problems of life and immortality which have attracted the minds of the profoundest thinkers since the earliest times in which men began to think at all. Sir Andrew Clark believed in the Christian faith with his whole soul. Now that he is dead it is fitting that this should be said of him. Standing in the midst of the ages, and bending his keen and powerful mind upon all the facts of the world and of life, of the past and the present, which he could marshall before him, he deliberately elected to call himself a believer in the cult of Christianity. By that he was content to stand. In that faith he lived and in that faith he died. This life so lived, those ideals so laboured after, those high hopes so trusted in, he has left as his legacy, not only to the medical profession, but to all men and women who live in their work and who make their work the one great object of their lives. Sir Andrew Clark has been a good and faithful servant of his generation; he has entered into his rest, and it is permitted to us to hope and believe that he has also entered into immortality and eternal blessedness.

The profession of which Sir Andrew Clark was so distinguished an ornament can ill spare him. He was a moderating personality. His presence made for forbearance, charity, and peace. He would think ill of no man unless facts were too strong for him. This charitable hopefulness often led him right when other men's too eager promptitude carried them to wrong judgments. His personality and his example will never be forgotten. He made the profession of medicine sweeter, more liberal, more just. Undoubtedly his memory and the recollection of his life and work will continue as influences long after his place is occupied by others; and medicine and the world will be better because Andrew Clark has lived and died.

